# Successful Treatment of Large B-Cell Lymphoma in a Child with Compound Heterozygous Mutation in the *ATM* Gene

**DOI:** 10.3390/ijms24021099

**Published:** 2023-01-06

**Authors:** Jakub Czarny, Marta Andrzejewska, Olga Zając-Spychała, Elżbieta Latos-Grażyńska, Agata Pastorczak, Kamila Wypyszczak, Aleksandra Szczawińska-Popłonyk, Izabela Niewiadomska-Wojnałowicz, Agnieszka Wziątek, Patrycja Marciniak-Stępak, Michał Dopierała, Jadwiga Małdyk, Katarzyna Jończyk-Potoczna, Katarzyna Derwich

**Affiliations:** 1 Faculty of Medicine, Poznan University of Medical Sciences, 61-701 Poznań, Poland; 2 Department of Pediatric Oncology, Hematology and Transplantology, Institute of Pediatrics, Poznań University of Medical Sciences, 60-355 Poznań, Poland; 3 Department of Pediatric Bone Marrow Transplantation, Oncology and Hematology, Wrocław Medical University, 50-556 Wrocław, Poland; 4 Department of Pediatrics, Oncology and Hematology, Medical University of Łódź, 91-738 Łódź, Poland; 5 Department of Pediatric Pneumonology, Allergy and Clinical Immunology, Institute of Pediatrics, Poznań University of Medical Sciences, 60-355 Poznań, Poland; 6 Department of Pathology and Clinical Immunology, Poznań University of Medical Sciences, 60-355 Poznań, Poland; 7 Department of Pathology, Medical University of Warsaw, 02-106 Warsaw, Poland; 8 Department of Pediatric Radiology, Institute of Pediatrics, Poznań University of Medical Sciences, 60-355 Poznań, Poland

**Keywords:** ataxia-telangiectasia, LBCL, DNA repair, rituximab

## Abstract

Ataxia-telangiectasia (AT) is a multisystemic neurodegenerative inborn error of immunity (IEI) characterized by DNA repair defect, chromosomal instability, and hypersensitivity to ionizing radiation. Impaired DNA double-strand break repair determines a high risk of developing hematological malignancies, especially lymphoproliferative diseases. Poor response to treatment, excessive chemotherapy toxicities, and the need for avoiding exposure to ionizing radiation make the successful clinical management of patients with AT challenging for oncologists. We describe the favorable outcome of the LBCL with *IRF4* rearrangement at stage III in a 7-year-old female patient diagnosed with AT. The patient was treated according to the B-HR arm of the INTER-B-NHL-COP 2010 protocol, including the administration of rituximab, cyclophosphamide, methotrexate, prednisone, etc. She presented excessive treatment toxicities despite individually reduced doses of methotrexate and cyclophosphamide. However, in the MRI there was no significant reduction in pathologic lymph nodes after three immunochemotherapy courses. Therefore, a lymph node biopsy was taken. Its subsequent histopathological examination revealed tuberculosis-like changes, though tuberculosis suspicion was excluded. After two following immunochemotherapy courses, PET-CT confirmed complete remission. From March 2022 onwards, the patient has remained in remission under the care of the outpatient children’s oncology clinic.

## 1. Introduction

Ataxia-telangiectasia (AT) is an inherited DNA double-strand break (DSB) repair disorder caused by biallelic *ATM* gene mutations. The gene encodes a serine-threonine kinase that controls the cell cycle, recognizes DNA double-strand breaks, and maintains genomic stability after exposure to DNA damage. The impaired function of ATM kinase results in the accumulation of genomic alterations that lead to the selective neurodegeneration of cerebellar Purkinje and granular layer cells.

The leading clinical symptomatology of AT includes progressively debilitating cerebellar ataxia with postural instability, ocular apraxia, dysarthria, and orolingual insufficiency, as well as extrapyramidal disorders, such as choreoathetotic movements, dystonia, and muscle tremor. The AT phenotype is also associated with chronic obstructive airway disease, interstitial lung disease, endocrine disorders, non-alcoholic fatty-liver disease, and cutaneous and systemic granulomatosis. The combined immune deficiency in AT encompasses impaired thymic T-cell development and lymphopenia, defective B-cell maturation with antibody deficiency and bone marrow failure, including a high risk of developing malignancies, especially non-Hodgkin lymphomas (NHL) and leukemias [1,2,3,4].

The definitive AT diagnosis requires the detection of biallelic pathogenic variants in the *ATM* gene. Coexisting common laboratory findings in AT comprise spontaneous rearrangements involving chromosomes 7 and 14 in the classical karyotype examination and an increased alfa-fetoprotein (AFP) concentration in the peripheral blood [1,3].

Patients with AT usually require physical rehabilitation, anti-infectious prophylaxis, a personalized vaccination schedule, immunoglobulin substitution (in case of hypogammaglobulinemia), and malnutrition prevention. Due to the susceptibility to malignancy, diagnostics of lymphoproliferative disease in case of the presence of suggestive for hematological cancer symptoms should be promptly started. However, treatment of malignancies in AT is challenging because of an increased risk of relapse and excessive chemotherapy-induced toxicities, including orointestinal mucositis, severe respiratory tract infections, feeding difficulties, and vincristine polyneuropathy. Therefore, the individualization of oncological treatment is almost always needed and involves reducing cytostatic dosages and adding immunotherapy and/or targeted therapies [2,5,6]. Rituximab added to the reduced-dose chemotherapeutic backbone in treating CD20-positive mature B-cell lymphomas in DNA repair disorders has been recommended [2]. Nevertheless, the beneficial effect of immunotherapy with rituximab in AT patients has not yet been proven in clinical trials. We describe successful treatment using the anti-CD20 antibody of primary resistant LBCL in the AT patient.

## 2. Case Presentation

### 2.1. Ataxia-Telangiectasia Diagnosis and Treatment

A seven-year-old female patient was born from the second, uncomplicated pregnancy of non-consanguineous parents without any significant family history of genetic diseases or malignancies. At birth, due to reduced muscle tone, the patient underwent rehabilitation.

When the patient started walking at the age of 12 months, she showed an unsteady gait with a broad base and waddling of the feet, which led her to be referred for a neurological consultation. By the age of two years, the patient suffered from recurrent respiratory tract infections and acute enteritis. She was hospitalized due to stomatitis with fever and rash, and infection with enterovirus, rhinovirus and human herpes virus 6 (HHV6) was proven. By then, the child had been vaccinated according to the mandatory vaccination schedule, including live BCG and measles–mumps–rubella (MMR) vaccines, without adverse effects following immunization. She also presented café-au-lait spots on the right lower leg and right subclavian region, a flat hemangioma on the skin if her back, an antimongoloid slant, a broad nasal root, and conjunctiva telangiectasias that occurred in the second year of life.

When the patient was two years of age, the immunological workup was performed. It showed very low IgG (130 mg/dL, N: 520–1360 mg/dL) serum level, complete IgA deficiency (<5 mg/dL, N: 45–135 mg/dL), and a markedly elevated serum IgM (up to 367 mg/dL, N: 46–190 mg/dL), corresponding with an unfavorable hyper-IgM phenotype. Flow cytometric peripheral blood (PB) immunophenotyping revealed a profound CD3+ T-cell lymphopenia, with low relative counts and absolute numbers of CD4+ T helper (Th) and CD8+ T cytotoxic/suppressor (Tc) cells. Within the Th cell compartment, a significant deficiency in the generation of CD3+CD4+CD31+RA+ recent thymic emigrants and CD3+CD4+CD27+RA+ naive cells was noted, accompanied by a very low naive-to-memory CD45RA+:CD45RO+ ratio equal to 0,03. In the B-cell compartment, increased relative counts and absolute numbers of immature non-switched B-cells were observable. The results of PB lymph cell flow cytometric analysis are displayed in Table 1.

In the course of microbiological studies, a streptococcal infection was found and treated with broad-spectrum antibiotic therapy. Hyperopia, astigmatism, and periodic convergent strabismus were also confirmed. Moreover, AFP concentration was elevated (93.82 ng/mL).

By the age of 4 years, she was hospitalized twice due to severe bacterial respiratory infections, during which she required an administration of oxygen therapy and broad-spectrum antibiotics treatment. Subsequently, the patient underwent dietary modifications and received antibiotic prophylaxis with azithromycin and inhaled anti-inflammatory treatment.

**Figure 1:**
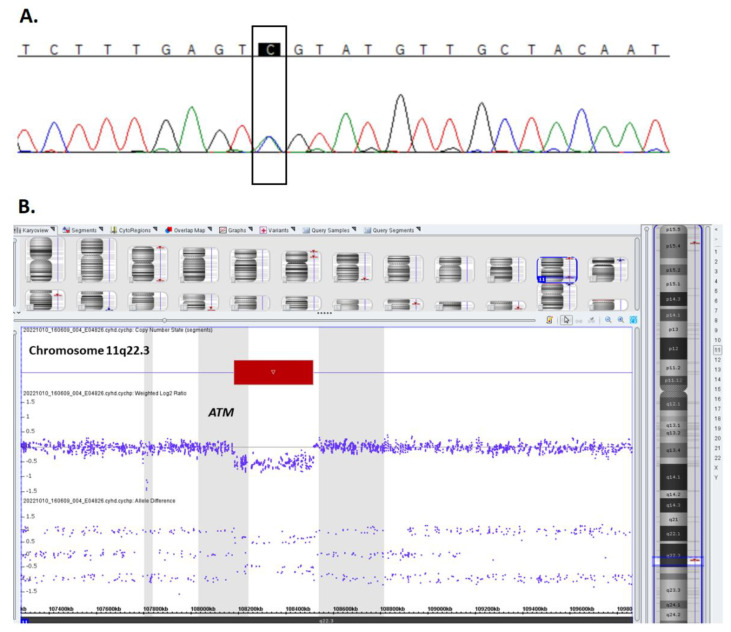
Pathogenic constitutional variants identified in the *ATM* gene in the proband. (**A**) Chromatogram displaying heterozygous missense mutation (NM_000051.3):c.434T>G within the *ATM* gene sequence (characteristic of the pathogenic sequence variant in Table S1). The electropherogram indicates the sequence of the anti-sense strand. (**B**) Chromosome microarray (CMA) data plot for chromosome 11 showing the germline monoallelic deletion of 11q22.3 encompassing exons 42–66 of the ATM gene. Deviations of probe Log2 ratios below 0 of the SNP probes on chromosome 11q22.3 cytoband indicate interstitial microdeletion identified in the genomic profile of the patient. The coordinates are in GRCh37/hg19.

Given the suspected diagnosis of ataxia-telangiectasia, the patient underwent genetic testing of the germline tissue. We performed whole *ATM* gene sequencing using the next-generation sequencing method, which revealed the presence of the pathogenic heterozygous c.434T>G, a p. Leu145Arg missense variant in the *ATM* gene (NM_000051.3). The presence of the variant was subsequently confirmed by direct sequencing. (Figure 1A).

Since the second sequence mutation within the *ATM* gene was not identified in the patient’s constitutional tissue, we searched for copy number abnormalities using a high-density single nucleotide polymorphisms array (SNP array). Array testing revealed the presence of monoallelic 333 kbp deletion at chromosome 11q22.3 (108180138–108513384) encompassing the 3′ end of the *ATM* gene (exons 42–66), *POGLUT3* and *EXP5* genes. (Figure 1B). Since each of the proband’s parent carries one heterozygous *ATM* variant we could confirm that the *ATM* defects are biallelic in the patient. Methods are described in the Supplementary Material S1 section [7,8,9,10,11,12,13,14,15].

### 2.2. Large B-Cell Lymphoma Diagnosis

**Figure 2:**
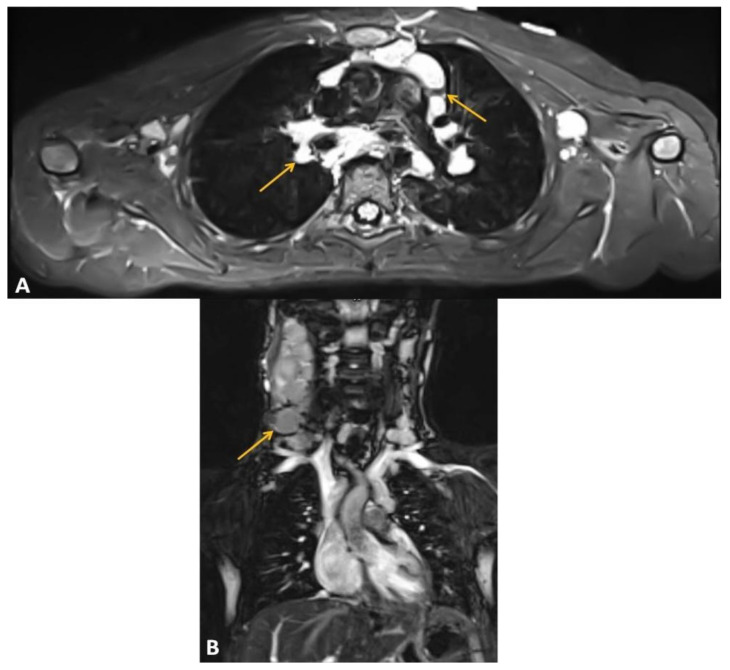
Chest MRI at the moment of LBCL diagnosis with the indication of carotic pathologic lymph nodes masses with arrows. (**A**) T2 STIR sequence axial. (**B**) T2 STIR sequence coronal.

**Figure 3:**
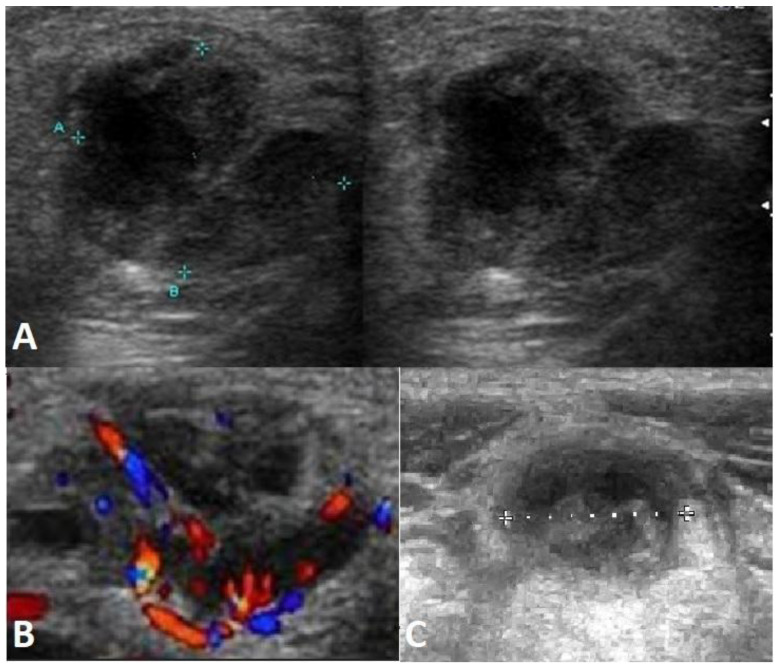
Ultrasound scans of pathological lymph nodes. (**A**) Lymph nodes with the potential breakup. (“A”, “B” indicate the borders of pathological lymph nodes) (**B**) Lymph nodes with incorrect vascular flow. (**C**) Inhomogeneous lymph nodes.

At the age of 6.5 years, significant lymphadenopathy of the right cervical, supraclavicular, and right submandibular lymph nodes occurred. Magnetic resonance imaging (MRI) (Figure 2) also showed enlarged lymph nodes in the mediastinum. Lymph node masses were detected in the mediastinal region from the superior thoracic aperture to Th7 level, in the anterior, middle, and posterior mediastinum; in both pulmonary hila and axillary cavities as well as in the lateral neck parts available in imaging. In the superior mediastinum, lymph nodes adhered to the posterolateral (left) circumference of the right brachiocephalic vein, surrounding the left brachiocephalic vein in its proximal part (without narrowing of vein lumen), adhering to the anterior and posterior of the vena cava superior circumference and anterior part of the aorta arch with its branches (without narrowing of their lumen). Lymph node masses model the right wall of the trachea unobtrusively without narrowing its lumen. The trachea was slightly relocated to the left by the lymph node masses on the right lateral neck parts (without the narrowing of their lumen visible in the MRI). In the cross-section, on the level of the superior edge of the aorta, lymph node masses sized 3.5 × 5.2 cm (ap × ds) and lymph node masses located subcarinally and right to the esophagus sized 1.8 × 3.7 cm (ap × ds) were observed. In both pulmonary hila, numerous lymph nodes up to 1.3 × 1.0 cm (ap × ds) on the right side and 1.4 × 1.0 cm (ap × ds) on the left side were imaged, as well as two lymph nodes of 0.3 × 0.8 cm and 0.5 × 1.0 cm to the left of the sternum. The biggest lymph nodes were in the axillas, on the right side 1.0 × 1.9 cm, on the left side 1.2 × 1.4 cm. The findings were verified with the ultrasound scans of pathological lymph nodes (Figure 3).

**Figure 4:**
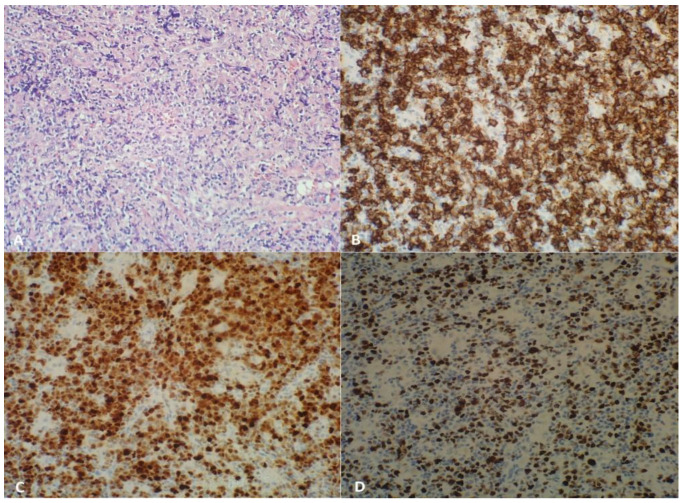
Neoplastic carotic lymph nodes detected at the age of 6.5 years: Large B-cell lymphoma with IRF4 rearrangement (×400). (**A**) Hematoxylin-eosin stain: atypical lymphoid cells (×400). (**B**) CD20-positive cells (×400). (**C**) MUM.1-positive cells (×400). (**D**) High count of Ki-67-positive cells (×400).

A biopsy of the right carotic lymph nodes was taken (Figure 4A–D). Microscopically, the lesion presented the structure erased by the proliferation of atypical lymphoid cells with the following immunophenotype: CD20 (+), MUM.1 (+), Bcl-6 (+); partially Bcl-2 (+) with CALLA (−), Cyclin 1 (−); CD30 (+) in moderately abundant diffused cells; CD21 (+) in the well-preserved network of dendritic cells; Ki-67 60–70%; EBV (−). Additionally, groups of epithelioid cells and vascular proliferation were seen. Stage III large B-cell lymphoma with IRF4 rearrangement was detected according to the WHO classification revised in 2016 [16].

NGS panel sequencing of LBCL biopsy material DNA confirmed a single homozygous mutation in the ATM gene c.434T>G (p.Leu145Arg); hence, in the same gene location as the germinal variant. Due to significant degradation of the DNA extracted from FFPE tumor tissue, exome sequencing results were of poor quality, which precluded reliable analysis of the presence of other somatic variants and their appropriate interpretation.

### 2.3. Large B-Cell Lymphoma Treatment

Treatment according to the B-HR arm of the Inter-B-NHL-COP 2010 protocol with dose reduction of methotrexate (without dose escalation) and cyclophosphamide was initiated, ultimately consisting of one cycle of pre-phase COP (Table 2), two cycles of R-COPADM (Table 3), and three cycles of R-CYM (Table 4).

Treatment was complicated by prolonged leukopenia and neutropenic fever (with an increase in inflammatory parameters, CTCAE Grade 3), as well as urinary tract infection (CTCAE Grade 3), which required broad-spectrum antibiotic therapy following the anti-infective prophylaxis. In addition, there were massive toxic lesions of the oral mucous membranes (CTCAE Grade 3). The patient required analgesic treatment, laser therapy, and intensive local care. The treatment was further complicated by recurrent thrombocytopenia and anemia (CTCAE Grade 3), requiring numerous transfusions of blood products. After the last treatment cycle, paroxysmal hypertension (CTCAE Grade 3) occurred and was treated with amlodipine.

After three cycles of chemoimmunotherapy with Pre-Phase COP, R-COPADM, and 1 R-CYM, a slight reduction in tumor-lesioned lymph nodes in imaging studies was observed in the treatment control. In comparison to the previous chest MRI, visible lymph node masses in the mediastinal region, both pulmonary hila, axillary cavities, and lateral neck parts available in the imaging were smaller or similar. In the right supraclavicular lymph node mass, a large soft tissue inflammation was observed in a cross-section of the altered soft tissue region with a total measurement of 60 × 25 mm (previously 69 × 28 mm). Periarterial mediastinal lymph nodes were as previously, whereas a paraaortic conglomerate presented with a size of 35 × 15 mm (previously 38 × 18 mm)—lymph nodes in the conglomerate were slightly smaller or similar. The right paratracheal superior lymph nodes were as previously, 16 × 8 mm, while the inferior were slightly smaller at 2 mm (previously 3 mm). The subcarinal lymph node mass was slightly smaller at 19 × 7 mm (previously 20 × 10 mm). A few pulmonary hila lymph nodes were imaged on the right, with the biggest one 10 × 5 mm as previously, and on the left, with the biggest one at 8 × 5 mm (previously 10 × 6 mm), and others were similar.

**Figure 5:**
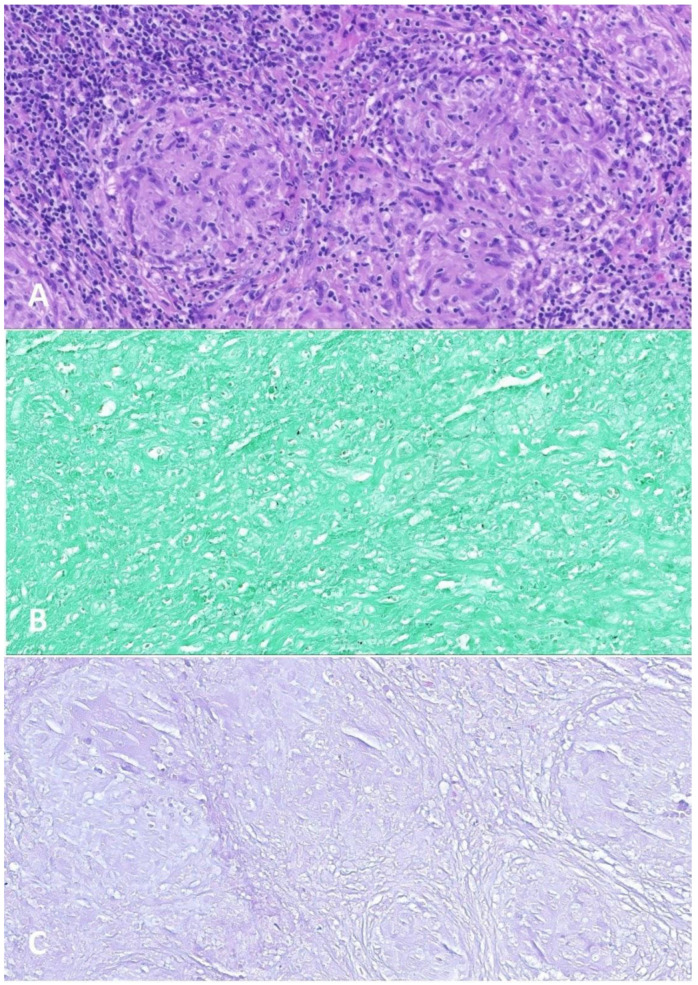
Small lymph nodes excised after Pre-Phase COP, 2 R-COPADM courses, and 1 R-CYM course. (**A**) Hematoxylin-eosin stain: granulomas (×200). (**B**) Grocott stain: negative for fungal organisms (×200). (**C**) Ziehl–Neelsen stain: negative for mycobacteria (×200).

After another two cycles of R-CYM in the histopathological control (Figure 5), bronchoscopy and PET-CT were undertaken despite the risk of tumor induction by X-rays due to the need to exclude the presence of tumor foci. The patient achieved complete remission. However, in the biopsy material, small lymph nodes were observed with a structure erased by tuberculosis-like granulomas without necrosis and festering traits. A large focus of necrosis observed with fine calcifications was surrounded by reactive cells. Bacilli and fungi stainings were negative. There was no Hodgkin/Reed–Sternberg cell, and CD30 staining was negative. Therefore, suspicion of tuberculous infection was excluded. The patient has remained under the care of the oncology clinic after treatment for 7 months.

## 3. Discussion

The described patient is a compound heterozygous carrier of pathogenic variants in the *ATM* gene and shows typical phenotypical features of AT, fulfilling the syndrome diagnosis criteria [4]. The age at lymphoma development was slightly lower as compared to the median age observed among individuals with AT. Still, the histopathological analysis of the tumor displayed one of the common malignant lymphoproliferations in AT, which is LBCL [1,2].

### 3.1. Large B-Cell Lymphoma Biology

LBCL with *IRF4* rearrangement derives from light-zone germinal center B-cells and usually carries aberrations within the genes encoding chromatin-modifying enzymes, PI3K signaling, and Ga-migration pathway components as well as structural variants affecting *BCL2*. Although this term is only a provisional lymphoma entity according to WHO classification and there is no particular biologic marker of this lymphoma, but it is possible to describe molecular characteristics of this heterogeneous group. Despite the lack of t(8;14), the Bcl2 protein is often expressed due to *BCL2* rearrangement present in between 25 and 50% of LBCLs with IRF4 rearrangement. In GC cells, co-expression of IRF4-MUM1, MUM1 (as itself), BCL6, and CD10 is observed, which is atypical for GC cells.

*IRF4* gene aberrations include gene fusions that arise from translocations, i.e., cryptic *IRF4::IGH* with *IGH* locus identified in most cases and strongly expressed *IRF4::MUM1*. Nonetheless, they are only present in some LBCLs; some cannot be detected routinely using FISH testing. This indicates a need of using more complex diagnostic genetic techniques, such as targeted next-generation sequencing (NGS). Furthermore, complex chromosomal aberrations (such as -7, -11q12.3-q25, +17p13) and aberrant somatic hypermutation (i.e., >4 mutations/case, including synonymous variants) are often present in LBCL with IRF4 rearrangement [17].

Mutations in LBCLs can also be found in the *CCND3* gene encoding cyclin D3 and NF-κB genes, such as *CARD11*, *CD79B,* and *MYD88*. Moreover, overexpression of downstream target genes of the NF-κB pathway is observed in LBCL. Ramis-Zaldivar et al. found that most *CARD11* mutations, exclusively found in cases with a diffuse growth pattern, are located in the coiled-coil domain, constitutively activating NF-κB, especially in adult diffuse large B-cell lymphoma. They also observed that all *CD79B* genetic changes contain Y197 hot spot mutation affecting the immunoreceptor tyrosine-based motif domain, reducing its negative regulation by the kinase LYN. What is more, rearrangement IG/MYC can also be detected in many LBCLs (35–90%, often of “double/triple-hit” (35–75% LBCLs) and rarely with IGH/MYC translocation). *TP53* mutations and high genetic complexity contribute to an unfavorable outcome. Moreover, between 35 and 75% LBCLs are described with high mutational status as “double-hit” lymphoma with the most common *MYC::BCL2* translocation or, rarely, *MYC::BCL6*, as well as “triple-hit” lymphoma with *MYC::BCL2::BCL6* translocation, or even “quadruple-hit” with *MYC::BCL2::BCL6::CCND1*. They also sometimes present rearrangement as *MYC::non-IG* [17,18,19,20,21,22,23,24,25].

LBCL usually affects the head and neck region in children and young adults and presents as a low-stage disease. Thus, the course of the disease substantially differs from excellent treatment results in non-syndromic patients compared to individuals with AT. Sato et al. described THRLBCL in multiple lymph nodes with bone metastases, which is exceedingly rare in children and AT patients. The abovementioned AT pediatric patient did not respond to the treatment (R-CHOP chemotherapy) and, before pre-transplant treatment, died of cardiac insufficiency and multiple organ failure induced by the rapid progression of the disease [26,27,28].

There is limited knowledge about the frequency and clinical impact of somatic *ATM* deletion in LBL. However, deletions at chromosome 11q22.3 encompassing the *ATM* gene are recurrent somatic alterations associated with poor prognosis in chronic lymphocytic leukemia. Its clinical significance may vary depending on the size of the deletion. Haploinsufficiency of a larger number of genes within 11q22.3 affects the proliferative advantage of leukemic cells and, therefore, strongly influences the patient’s outcome [29]. Additionally, somatic *ATM* deletions were also associated with reduced survival in mantle cell lymphoma [30].

### 3.2. Large B-Cell Lymphoma Treatment in the Presence of Ataxia-Telangiectasia

The initial response of our patient to oncological treatment can be described as partial with concomitant severe chemotherapy-induced side effects. Individuals with inherited DNA repair defects, including AT, usually show more severe toxicities as compared to non-syndromic individuals [1]. In the described case, all toxicities were manifested as CTCAE Grade 3 [5]. Therefore, concerning oncological treatment, it is recommended to reduce cytostatic dosages (especially of methotrexate, including intrathecal injection), and extend the role of immunotherapy and targeted therapies, including CAR-T therapy in relapsed/recurrent acute lymphoblastic leukemia and NHLs that dominate in malignancies of AT patients [2,31]; although, we followed the recommendations for the management of lymphoma in DNA repair disorders and also treated the side effects of the therapy.

### 3.3. Follow-Up of Large B-Cell Lymphoma Treatment in the Light of the Presented Case

Furthermore, in the described case, primary complex immunodeficiency led to atypical infection, causing lymph node enlargement, possibly simultaneously with the development of LBCL. It indicates the need for a histopathological study of lymph node enlargement in patients with AT. Pathomorphological examination of excised carotic lymph nodes enabled to ascertain of the actual infiltration of lymphoma in combination with PET-CT needed to exclude the presence of tumor foci. It was assessed that the possible benefits from lymphoma presence verification outweigh the risk of cancerogenesis due to the increased cell radiosensitivity. However, each decision regarding X-ray radiation application in DNA repair deficiency disorder patients should be considered individually [32]. This case shows the need for pathomorphological verification of the reason for lymphadenopathy in AT pediatric patients. Immunodeficiency in these patients and increased susceptibility to malignancy can lead to a similar image of enlarged lymph nodes upon physical examination and in ultrasound or MRI images. Therefore, accurate elucidation of the diagnostic difficulties is crucial for an appropriate treatment of an AT patient.

## 4. Conclusions

A thorough clinical evaluation by a multidisciplinary team, as well as in-depth immunological diagnostics, including assessment of humoral and cellular immune responses [33], in conjunction with molecular genetic testing, leads to the diagnosis of AT. Immunoglobulin replacement therapy and antibiotics are the mainstays of prophylaxis against infections in AT. Due to the particular susceptibility of AT patients to malignancies, oncological surveillance must be highlighted in every child affected with AT. The addition of immunotherapy in selected cases may substantially increase treatment efficacy and contribute to long-term survival. Moreover, in-depth molecular knowledge about LBCL enables better stratification of patients and application of appropriate treatment. The off-protocol diagnostics method to investigate the treatment efficacy is necessary in case of any doubts about the course of cancer in AT patients because of co-occurring immunodeficiency.

## Figures and Tables

**Table 1:** The PB lymphocyte subsets in the patient aged 2-years-old, at the time of AT diagnosis.

PB Lymphocyte Subsets	Results	Reference Values
WBC	5600 cc	
Lymphocytes CD45+/SSC low	35.0%, 1960 cc	29.6–69.2%, 2300–6900 cc
B CD19+	35.0%, 700 cc	14.1–28.5%, 400–1700 cc
Transitional B CD19+CD38+IgM++	4.3%, 30 cc	3.1–12.3%, 20–200 cc
Mature naïve B CD19+CD27-IgD+	36,2%, 253 cc	54.0–88.4%, 280–1330 cc
Non-switched memory B (MZL) CD19+CD27+IgD+	39.4%, 276 cc	2.7–19.8%, 20–180 cc
Switched memory B CD19+CD27+IgD-	22.1%, 155 cc	4.7–21.2%, 20–220 cc
Immature B CD19+CD21lo	44.4%, 311 cc	4.1–24.4%, 20–230 cc
Activated B CD19+CD38loCD21lo	4,6%, 11 cc	1.7–5.4%, 10–60 cc
Plasmablasts CD19+CD38++IgM-	0.3%, 2 cc	0.6–4.0%, 5–10 cc
T CD3+	17.0%, 340 cc	52.0–92.0%, 850–4300 cc
T helper CD3+CD4+	9.0%, 180 cc	25.0–66.0%, 500–2700 cc
T suppressor/cytotoxic CD3+CD8+	7.0%, 140 cc	9.0–49.0%, 200–1800 cc
CD4+/CD8+	1.29	1.5–2.5
CD4+CD45RA+/CD4+CD45RO+	0.03	>1.0
Recent thymic emigrants CD3+CD4+CD45RA+CD31+	2.2%, 4 cc	37.0–100%, 190–2600 cc
Naïve T helper CD3+CD4+CD45RA+CD27+	1.8%, 3 cc	52.0–92.0%, 300–2300 cc
Central memory T helper CD3+CD4+CD45RA-CD27+	97.2%, 167 cc	15.0–56.0%, 160–660 cc
Effector memory T helper CD3+CD4+CD45RA-CD27-	4.7%, 8 cc	0.3–9.0%, 3–89 cc
Terminally differentiated memory T helper CD3+CD4+CD45RA+CD27-	0.9%, 2 cc	0.0–1.2%, 0–16 cc
Follicular CXCR5+ T helper CD3+CD4+CD45RO+CD185+	48.2%, 84 cc	6.0–72.0%, 13–170 cc
Regulatory T helper CD3+CD4+CD25++CD127-	1.3%, 2 cc	3.0–17.0%, 39–150 cc
Naïve T suppressor/cytotoxic CD3+CD8+CD27+CD197+	12.2%, 17 cc	19.0–100%, 53–1100 cc
Central memory T suppressor/cytotoxic CD3+CD8+CD45RA-CD27+CD197+	12.2%, 17 cc	1.0–9.0%, 4–64 cc
Effector memory T suppressor/cytotoxic CD3+CD8+CD45RA-CD27-CD197-	12.8%, 18 cc	10.0–55.0%, 24–590 cc
Terminally differentiated T suppressor/cytotoxic CD3+CD8+CD45RA+CD27-CD197-	12.2%, 17 cc	6.0–83.0%, 25–530 cc
NK CD3-CD45+CD16+CD56+	41.0%, 820 cc	2.0–25.0%, 61–510 cc

Notes: cc-cells/mm^3^.

**Table 2:** Treatment according to the B-HR arm of the Inter-B-NHL-COP 2010 with dose reduction of methotrexate and cyclophosphamide, without methotrexate dose escalation—Pre-Phase COP.

Day	1	2	3	4	5	6 = Day 2 of 1st R-COPADM	7
Vincristine	x						
Prednisone	x x	x x	x x	x x	x x	x x	x x
Cyclophosphamide	x						
IT MTX and HC	x						

Notes: Vincristine 1.0 mg/m^2^ (max single dose 2.0 mg/m^2^) as IV bolus; Prednisone 60 mg/m^2^/day (divided into bid doses) orally; Cyclophosphamide 300 mg/m^2^/dose as an infusion over 15 min; IT drugs Methotrexate (MTX) and Hydrocortisone (HC) 15 mg + 15 mg intrathecal (IT) injection. X means the administration of respective medication.

**Table 3:** Treatment according to the B-HR arm of the Inter-B-NHL-COP 2010 with dose reduction of methotrexate and cyclophosphamide, without methotrexate dose escalation—R-COPADM.

Day	1	2	3	4	5	6
Rituximab	x					
Vincristine	x					
Prednisone	x x	x x	x x	x x	x x	Tail to zero over 3 days
Methotrexate	x					
Folinic acid		xxxx	xxxx	xxxx		
Cyclophosphamide		x x	x x	x x		
Doxorubicin		x				
IT MTX and HC		x				x

Notes: Rituximab 375 mg/m^2^; Vincristine 2.0 mg/m^2^ as IV bolus; Prednisone 60 mg/m^2^/day (divided into bid doses) orally; Methotrexate 1.0 g/m^2^ in 500 mL/m^2^ dextrose 5% as intravenous infusion; Folinic acid 15 mg/m^2^ orally every 1 h until MTX level is below 0.15 mmol/L (1.5 × 10^−7^ M); Cyclophosphamide 200 (1st R-COPADM)/250 (2nd R-COPADM) mg/m^2^/dose every 12 h as an infusion over 15 min. Continue hydration at a rate of 3000 mL/m^2^/day until 12 h after the last dose of cyclophosphamide; Doxorubicin 60 mg/m^2^/day in 1-h infusion; IT drugs Methotrexate and Hydrocortisone 15 mg + 15 mg IT injection. X means the administration of respective medication.

**Table 4:** Treatment according to the B-HR arm of the Inter-B-NHL-COP 2010 with dose reduction of methotrexate, without methotrexate dose escalation—R-CYM.

Day	1	2	3	4	5	6	7
Rituximab	x						
Methotrexate	x						
Folinic acid		xxxx	xxxx	xxxx			
Cytarabine		x					
IT MTX		x					
IT HC		x					x
IT ARA-C							x

Notes: Rituximab 375 mg/m^2^; Methotrexate 1.0 g/m^2^ in 500 mL/m^2^ dextrose 5% as intravenous infusion over 3 h; Folinic acid 15 mg/m^2^ orally every 6 h until MTX level is below 0.15 mmol/L (1.5 × 10^−7^ M). This begins at 24 h from the start of the methotrexate infusion; Cytarabine 100 mg/m^2^ in 1000 mL/m^2^ dextrose saline as an infusion over 24 h; IT Methotrexate 15 mg by IT injection; IT Cytarabine 30 mg by IT injection; IT Hydrocortisone 15 mg by IT injection. X means the administration of respective medication.

## Data Availability

Not applicable.

## Supplementary Materials

The following supporting information can be downloaded at: https://www.mdpi.com/article/10.3390/ijms24021099/s1.
