# Supplementary material for: Successful Treatment of Large B-Cell Lymphoma in a Child with Compound Heterozygous Mutation in the ATM Gene
Source: Int J Mol Sci. 2023 Jan 6;24(2):1099. doi: 10.3390/ijms24021099 (PMC9866559; doi:10.3390/ijms24021099)
Supplement: Supplementary file 1 [file ijms-24-01099-s001.zip › ijms-2076241-supplementary.pdf]

International Journal of
Molecular Sciences

MDPI

Supplementary Material S1.

Methods

DNA isolation

Genomic DNA was extracted from peripheral blood samples at remission using the QIAamp DNA Blood mini kit (QIAGEN, Hilden, Germany. The concentration and quality of isolates were determined by ultraviolet spectrophotometry (NanoDrop 8000, Thermo Scientific, Waltham, USA).

Microarray analysis

Copy-number variation analysis was performed using CytoScan HD array (Applied Biosystems, Thermo Fisher, Waltham, MA). All laboratory procedures were carried out according to the manufacturer's protocols as described previously [7]. Chromosome Analysis Suite v 4.2 software (ChAS, Thermo Fisher Scientific, Waltham, MA) was used for the analysis of copy number, loss of heterozygosity (LOH), mosaicism, and genotype calls.

Next Generation Sequencing

Next generation sequencing (NGS) was carried out with TruSightOne Sequencing Panel, on NextSeq550 machine, Illumina in the process of 300 bp paired-end run using Mid Output Kit (Illumina). The data analyses of the target regions were performed using Burrows-Wheeler Aligner Genome Alignment Software and the GATK Variant Caller algorithms and mapped to the human genome reference sequence GRCh37/hg19. [8,9] The results were analyzed using Variant Studio v. 3.0 (Illumina) and Integrative Genomics Viewer v.2.3. [7] The pathogenicity of the revealed changes was estimated based on standard bioinformatics tools, such as: Mutation Taster, SIFT or PolyPhen-2 [10–12] and using several databases, such as ClinVar, HGMD and OMIM [13-15].

Direct sequencing

For presence of the pathogenic c.434T>G, p. Leu145Arg variant in the *ATM* gene (NM_000051.3). detected in NGS was confirmed using direct sequencing. Standard PCR conditions were used with the primers specifically designed to analyze the mutation using NetPrimer software (forward primer: 5'-TAGTTGCCATTCCAAGTGTC-3'; reverse primer: 5'-AAACTGTCAGGTCACTTGGG-3'). Products were sequenced on ABI3130 4-capillary sequencer (Thermo Fisher Scientific) and the results were analyzed using Sequencher v. 5.0.

Table S1. Characteristic of the pathogenic heterozygous *ATM* sequence variant identified in the proband.

**Gene**	*ATM*
**Chromosome**	11
**Cytoband**	11q22.3
**Position**	108106499
**Ref sequence**	T
**Alt sequence**	G
**HGVS**	NM_000051.3:C.434T>G
**Protein**	NP_000042.3:p.Leu145Arg
**Consequence**	missense variant
**Frequency (GnomAD_exome)**	No data available
**GERP# score**	5.6199
**DANN* score**	0.9975
**MutationTaster score**	0.9999
**ACMG classification**	Variant of Uncertain Significance (PP3, PM2)
**Total RD**	495/1093

# Genomic Evolutionary Rate Profiling - is a conservation score calculated by quantifying substitution deficits across multiple alignments of orthologues using the genomes of 35 mammals. It ranges from -12.3 to 6.17, with 6.17 being the most conserved.

* DANN is a pathogenicity scoring methodology, which is based on deep neural networks. The value range is 0 to 1, with 1 given to the variants predicted to be the most damaging.

RD – read depth
